# Supplementary material for: Are carbon emissions trading and green financial instruments synergistic? -Comprehensive quantitative research based on content analysis
Source: PLoS One. 2024 Mar 7;19(3):e0298601. doi: 10.1371/journal.pone.0298601 (PMC10919601; doi:10.1371/journal.pone.0298601)
Supplement: S1 Data — (ZIP) [file pone.0298601.s001.zip › Data/地级市Z_F工作报告.zip!/║┌┴·╜¡/╝╤─╛╦╣╩╨/└┤╫╘╣1⁄2╓┌║┼í╛┬φ┐╦╩2╛▌═°í┐_2012.docx]

各位代表：

现在，我代表市人民政府向大会报告工作，请予审议，并请各位政协委员和列席会议的同志提出意见。

一、过去五年的工作回顾

　　过去的五年，全市人民在省委、省政府和市委的坚强领导下，牢牢把握科学发展这个主题，励精图治，开拓进取，全市经济社会发展呈现出喜人局面。

　　五年来，综合实力显著提升。预计全市地区生产总值从276.4亿元增加到600亿元，增长1.17倍，年均增长15.1%；财政总收入从13.9亿元增加到65亿元，增长3.7倍，年均增长36.4%；财政一般预算收入从6.2亿元增加到31.5亿元，增长4倍，年均增长38.2%；粮食产量从70亿斤增加到146亿斤，增长一倍，年均增加15.2亿斤；规模以上工业增加值由31.46亿元增加到90亿元，增长1.86倍，年均增长23.4 %；社会消费品零售总额由101.5亿元增加到245亿元，增长1.41倍，年均增长19.3%；固定资产投资由60.7亿元增加到276.7亿元，增长3.56倍，且五年累计完成896.8亿元，是前一个五年的4.4倍；进出口总额由10亿美元增加到36.1亿美元，增长2.4倍，年均增长27.8%。建设产业园区10个，完成征地面积19.5平方公里，完成项目投资64.8亿元。其中市高新区已晋升为省级园区。各县（市）在全省综合排名中，位次均有较大晋升。

　　五年来，区域中心城市形象日益彰显。以成功举办第十二届省运会和第八届省少数民族运动会为标志，区域文化、教育、体育、卫生中心的引领作用显著增强，“伊玛堪”被联合国教科文组织列入“急需保护的非物质文化遗产名录”，填补了我省空白；一大批文艺精品先后在国家、省赛事中获得佳绩；各级各类教育资源得到进一步优化，全面实现了免费九年义务教育，普通高中高考捷报频传；“快乐舞步”等群众性体育活动蓬勃开展。独立开展医学新技术、新项目84项，其中9项处于全省领先地位，13项填补省内空白。整体医疗水平不断提高，医疗条件显著改善，面貌一新。批发零售企业发展到5200家，一大批大型综合商场及专业批发大市场建成投入使用。口岸贸易日益繁荣，同江、抚远口岸过货能力显著提升。佳木斯国际航空港通过了国家验收，并开通佳木斯至首尔、佳木斯至哈尔滨航线，佳木斯至上海航线由每周2班增加到6班，带动旅游业和项目建设加快发展。成功承办两届中俄（佳木斯）农机产品展销洽谈会。商贸、物流、旅游产业发展迅速，第三产业增加值预计实现267亿元，增长1.15倍，年均增长13.7%。市区累计85%的主干道和65%的区街巷道得到维修改造，新建主次干道41条，区街巷道221条，新扩建长安路立交桥、光复路立交桥等桥梁10座。公园面积达到708公顷，建成区绿化覆盖率达到40.3%。建设各级公路6160公里，全市乡镇、行政村全部通达等级公路，佳木斯至双鸭山、七台河、同江，建三江至虎林等高速公路及松花江富绥大桥、前抚铁路均建成通车，对周边区域辐射作用强劲。

　　五年来，主动破解难题，发展活力显著，人民生活持续改善。国企改革攻坚任务基本完成，妥善安置职工8万余名，4万余名国有困难企业退休职工纳入基本医疗保险，6021名大集体职工和2.1万名“五七工”纳入城镇社会养老保险统筹，清欠职工款3亿元，化解并轨国企历史债务26亿元。争取社保资金57.1亿元，创业培训6.69万人，实现城乡新就业68.82万人次，分别比上一个五年增长1.34倍、1.46倍和2.5倍，城镇登记失业率始终控制在4.5%以下。全市征收房屋98915户、584万平方米，改造旧小区86个、73万平方米，改造棚户区696.2万平方米，建设廉租房、公租房19186套，有效改善了人民群众的居住环境。全市城镇养老、医疗、失业、工伤、生育参保人数分别达到了23.55万人、76.45万人、18.94万人、20万人、8.69万人，分别比2006年增加1.1倍、1.27倍、3.3 %、1.1倍和3.8倍，新农保参保35.36万人。企业退休人员养老金标准人均增加627元，失业救济金标准由214元提高到400元，城镇职工住院综合报销比例由71%提高到83%，城市低保标准由月人均160元提高到280元，农村低保由年人均800元提高到1450元。实施了医疗、教育、供热、扶贫解困等16项专项救助。城镇居民人均可支配收入由7791元增加到13892元，年均增长12.3%；农民人均纯收入由3630元增加到8600元以上，年均增长18.8%。集中供热普及率达到75%，天然气普及率达到53%。生活污水达标率85%，空气质量二级以上天数345天。市区取消中巴车运营，公交车辆状况得到了改善。

　　五年来，政府执政能力不断提高。全面实施行政审批、政府投资、重大决策、行政效能监察、“三重一大”、政务公开等制度，政府的执行力和公信力不断提高。政府性资金全部纳入审计监督范围，有效提高了公共资源配置使用效能。精简审批程序223项。办理人大代表建议602件、政协委员提案1339件，办结率达100%。各种信访积案办结率95%，群众合理诉求基本解决。清理整顿建设项目104个，追缴税费6574万元。法制型、学习型、节约型、服务型政府建设不断加强，政府工作效率不断提高。

　　各位代表，2011年是本届政府施政的最后一年，按照“十二五”发展规划，年初确定的各项目标任务已全面完成，实现了“十二五”发展的良好开局。全市地区生产总值比上年（以下同比）增长14%；财政总收入同比增长29.5%；财政一般预算收入同比增长51%；社会消费品零售总额同比增长17.9%；固定资产投资同比增长30%；进出口总额同比增长 18.3%。

　　2011年，强力推进产业项目建设。全市开工千万元以上产业项目340项，亿元以上项目70项，分别比上年增长126.7%、150%；完成投资140亿元，同比增长37.3%。开工建设了常发佳联收获机械等装备制造业项目48个；中储粮油脂公司20万吨大豆仓储加工等农产品深加工项目71个；北方水泥公司4500吨新型干法熟料生产线等资源优势项目42个。升级改造了在线低辐射镀膜玻璃生产线等新材料项目35个；多多药业克林霉素、葵花药业异地新建等生物产业项目8个。全市销售收入超亿元企业达到70户，规模以上工业实现增加值同比增长26%。产业园区建设初具规模，其中市高新区农业装备园、富锦绿色食品园被列为省市共建园区，为项目落地，增强发展后劲奠定了基础。金融业新增贷款余额100亿元，达440.8亿元，增长28.6%；存款余额达641.7亿元，增长2.6%，对地方经济的支持力度显著增强。

　　2011年，产业结构进一步优化。三次产业比重由2010年的28.6：26.1：45.3调整为26.1：29.4：44.5，第二产业增强。农业发展质量提高，以“千万亩、百亿斤”水稻种植为目标，新增水稻面积213.7万亩，全市目前达到659万亩，增量位居全省第一位。玉米种植面积达到583万亩。绿色食品种植面积756.8万亩，实物总量245万吨，同比增长3.5%和7.5%。蔬菜种植面积58.6万亩，比上年增加9.4%。以育秧大棚利用为切入点，积极探索提高土地利用效率。预计畜牧业总产值可实现77亿元，增长19.4%，占农业总产值28.5%。新申报地理标志6个，占全省五分之一。与区域内省属20个农场、2个省属林业局开展共建，“三代”作业面积470万亩，共建城镇道路90.2公里。高起点打造了现代化农业“三个百万亩示范带”，一个“集中展示区”。新农村建设步伐加快，16个中心镇、32个中心村建设取得新进展，18个村镇被省评为四星级以上示范村镇。积极推进高新技术产业发展，全市国家级高新技术企业已达到13家，省级高新技术企业43家，省级创新型企业16家，新认定省级高新技术产品51项，高新技术产值达160.8亿元，同比增长40.3%。发明专利申请量、受权量同比分别增长25%和100%。国家级防爆电机工程中心建成。率先在全省建立了小企业服务平台。

　　2011年，对外开放取得了新成果。对外交往进一步扩大，与国外开展友好联系的城市增加到近40个。意大利阿维利诺省企业、法国两大糖业集团等与我市开展合作。成功举办第二届中俄（佳木斯）农机展，吸引俄罗斯、乌克兰、加拿大、日本、韩国等国和国内25个省市5万多人参会，总成交额10.78亿美元。在央企合作交流、港澳招商周、哈洽会等全省三次重大经贸活动中共签约项目20个，签约总额233.6亿元，且全部开工建设。航空口岸进出港人数达21.3万人次，增长11.5%。黑瞎子岛开放开发吸引了大量海内外游客。预计全市全年招商引资到位资金200亿元，同比增长38%，实际利用外资达到1.45亿美元，同比增长28%。

　　2011年，城乡面貌焕然一新。以国家实施保障性安居工程建设为契机，全力推进城乡住宅改造及配套基础设施工程，全市征收房屋65742户、383.28万平方米，其中政府投入征收房屋资金7.8亿元，是上一年的2倍。市区拆除违建24万平方米，是上一年的14倍。改造农村泥草房12040户，面积103.5万平方米。全市新开工住宅面积591万平方米。解决了149栋72万平方米“弃管楼”问题。完成中心城区巷道建设66条，开工建设主次干道21条段，其中有14条段完工。狠抓建设工地管理，规范施工过程，三废出社区行动扎实推进。以创建国家园林城为牵动，充分利用冬春两季，对新建道路及原有街道行道树进行栽植，并植树造林31.82万亩，建设绿色通道340公里。西区污水处理厂建成投入使用。郊区湿地大型灌区农田退水污染防治工程被列为国家示范工程。“百镇”建设取得了新进展，创建国家级生态乡镇1个，省级7个。

　　2011年，发展成果惠及更多的人民群众。大力推进中小学校舍安全加固工程，开工237栋，面积54.6万平方米。新建、改扩建公办幼儿园29所，竣工交付使用28所，幼儿入园率84.7%。普通高中高考重点本科上线人数首破800人。解决了市孤儿院房舍漏雨和不保温问题。创业培训1.16万人，实现创业带动就业5.61万人。医疗救助标准提高到1—1.5万元，大病补偿最高支付限额超过5万元，均位居全省前列。新农合参合农民102.27万人，参合率99.48%。基层医疗卫生机构提供城乡基本公共卫生服务能力有了提高。全市城镇养老保险新增参保9389人，新农保覆盖面达到83%。建设了储气量150立方米的LNG液态燃气储备罐两座，增加供气能力18万立方米，解决了冬季气源不足问题，城区新装天然气用户1.8万户。针对煤电价格倒挂，市政府安排1400万元补贴市区东、西电厂运行，以保持居民热费价格稳定。乡镇自来水普及率达到76.5%，农村自来水普及率达到65.8%。东风兴电等两个社区及街道办事处被命名为“全国和谐社区建设示范单位”，社区服务网点达到2680个。八类主要刑事案件五年来首次呈全部下降局面，社会治安公众满意率达到93.8%，10个县（市）区全部进入省“平安县、区”行列。全国双拥模范城实现“七连冠”。构建从乡到市“三位一体大调解体系”，建立县（区）、市领导信访例会、督查督办机制，信访群众的合理诉求得到有效解决，信访案件大幅下降。安全生产形势总体稳定，应急机制进一步完善。高度重视民族宗教工作，物价、食品药品、工商、质检等市场监管力度加大，人口计生奖励政策全面落实，妇女儿童、老年人、残疾人权益得到进一步保障，防灾减灾、拥军优属、国防人防、外事侨务、统计咨询、红十字会和社会慈善等各项社会事业取得新进展。

　　回顾走过的历程，我们深深感到：开拓创新，勇于实践，奋力推进产业项目建设，实现大发展、快发展就有了前提保障；转变发展方式，调优产业结构，实现大发展、快发展就有了坚实基础；扩大开放，深化改革，实现大发展、快发展就有了旺盛活力；提升城市功能，管理有序到位，实现大发展、快发展就有了能量载体；加强社会建设，致力改善民生，实现大发展、快发展就有了不竭动力。只有把为民谋利作为政府工作的根本，实现好、维护好人民群众利益，就能够得到人民群众的拥护，就能够团结和调动一切积极因素，共同推进佳木斯发展的伟大进程！

　　各位代表，我们取得的成绩来之不易，积累的经验和体会弥足珍贵。这是省委、省政府和市委正确领导的结果，是市人大和市政协关心支持的结果，是几任班子打下良好基础的结果，是全市人民共同奋进的结果，是社会各界鼎力支持的结果。在此，我代表市人民政府，向所有为全市改革发展稳定作出贡献的同志和支持佳木斯发展的各界朋友表示衷心的感谢！向离退休的老同志表示深深谢意！向在佳木斯创业的投资者和建设者致以崇高的敬意！

　　在肯定成绩的同时，我们也清醒地看到经济社会发展中存在的矛盾和问题。主要是固定资产投资总量低，产业项目占固定资产投资比重小，特别是缺少龙头和领军型大项目；结构性矛盾依然突出，精深加工业和新兴产业比重不大，经济发展的质量和效益有待进一步提高；县域经济总量不大，拉动作用不明显；财政支出压力大，保障和改善民生的能力弱；一些历史欠账及深层次矛盾导致群众不满意问题还没有彻底解决好；政府自身建设有待进一步加强，经济社会发展环境有待进一步优化。对此，我们要高度重视，采取更加有力的措施，认真加以解决。

　　二、未来五年的主要任务

　　今后五年，政府工作的指导思想是：高举中国特色社会主义伟大旗帜，以邓小平理论和“三个代表”重要思想为指导，按照市委十二次党代会确定的“以科学发展为主题，以加快转变经济发展方式为主线，以大项目建设为突破口，突出四大优势，全面完成九大战略任务”的部署，扎实推进经济建设、政治建设、文化建设、社会建设以及生态文明建设，努力改善民生，为建设富裕、文明、和谐、幸福佳木斯而努力奋斗。

　　今后五年，我市经济社会发展的战略目标是：到2016年，地区生产总值超过1200亿元，年均增长13%；全社会固定资产投资突破1000亿元，年均增长30%以上；财政一般预算收入达到75亿元，年均增长20%；规模以上工业增加值220亿元，年均增长20%；社会消费品零售总额达到500亿元，外贸进出口总额实现90亿美元，年均增长20%；单位GDP能耗累计降低16%，主要污染物排放总量累计下降5%，森林覆盖率达到17.2%；人口自然增长率控制在5‰以内，城镇登记失业率控制在4.5%以内；城镇居民人均可支配收入达到25500元，农民人均纯收入达到15000元。

　　——突出农业优势，推动现代农业大发展。推进“千万亩”水稻工程，新建、续建临江、三村等8大灌区，建成高产、稳产水稻生产区。到2016年，全市水稻面积发展到1000万亩，产量100亿斤以上。大力推广以“八化”为标准的示范区建设，提高各种作物单产，增加总产。鼓励多种经营，“打绿色牌，走特色路”，形成规模化特色经济。围绕大宗优势农产品，打造高标准的农业产业化龙头企业群，延伸产业链，提升竞争力。继续推动中心村、小城镇建设，引导农民在增收同时转变生活方式，建设一批经济强镇、旅游名镇、生态特色小镇，加快城乡一体化进程，推动城镇化快速发展。

　　——突出工业基础优势，推动产业大提升。把招商引资、产业项目建设、园区建设、扩大开放、矿产资源开发作为我市加快崛起的关键措施。引进有助形成产业高地，成为新经济增长点的战略投资。五年累计国内招商引资到位资金1700亿元，实际利用外资10亿美元以上。大项目建设突出产业项目，产业项目突出工业项目，工业项目突出财政贡献率，并注重产业配套聚合。着力提高产品创新能力和品牌创造能力。五年建设重点产业项目450个。

　　——突出沿边开放优势，推动对俄大开放。紧紧抓住国家、省有关沿边地区开放开发政策机遇，放大我市沿边开放优势。充分发挥同江、抚远对俄开放带动作用，加快对俄经贸科技合作方式调整，适应俄加入WTO政策变化，推动建设进出口产业加工园区、境外加工产业园，调整进出口产品结构，扶持外贸龙头，最大限度地发挥我市对俄合作桥头堡和枢纽站的作用。强化口岸建设，打造水、陆、空立体开放格局。到2016年，口岸吞吐能力力争全省第一位。

　　——突出中心城市优势，推动区域经济大融合。加强省东部城市群区域内各大经济单元合作共建，强化经济区城市协作。以构建现代商贸流通体系为重点，推进具有物流集散、批发贸易、信息发布、电子商务、金融服务等功能的市场建设，提升农机、服装、汽车等大型展会水平。依托教育、卫生、文化、体育等优势资源，培育壮大名校、名院、名团、名队，打造教育最好、卫生领先、文化先进、群体活跃的三江名城。依托大学、职教中心和科研院所及重点实验室，培育产学研资介结合的科教创新体系，打造省东部城市发展智力科技支撑平台。拓展城市发展空间，完善城市功能，提升中心城市综合承载能力，提高城乡居住水平，全力打造国际化滨水园林城市。

　　三、2012年的主要工作安排

　　全市经济社会发展的主要预期目标是：地区生产总值增长13 %，规模以上工业增加值增长20 %，全社会固定资产投资增长35 %，社会消费品零售总额增长17 %，外贸进出口总额增长20 %，财政一般预算收入增长20 %，城镇居民人均可支配收入增长13%，农民人均纯收入增长13%。城镇登记失业率控制在4.5 %以内，人口自然增长率控制在5 ‰以内，节能减排完成省下达的目标任务。

　　上述目标的确定，充分考虑到国内外经济形势的变化，注重了与“十二五”规划目标相衔接，也注意与省政府目标任务相一致，更顺应全市人民希望快发展、惠民生的真诚期待。实现这个目标任务，必须坚持统筹兼顾，突出重点，在财政资金安排上既要有保有扶有控有压，又要尽力而为，量入为出。用于保民生的钱必须留足，用于发展的钱主要靠市场运作，靠盘活资产，利用好各种资源效益。我们坚信，通过全市人民的共同努力，预期的经济社会发展目标一定能够实现。

　　为实现上述目标，着力抓好九个方面工作：

　　（一）大力推进招商引资，加快产业项目建设。全市计划新开工千万元以上产业项目200项，谋划产业项目300项，产业项目投资超过200亿元，增长40%以上。国内招商引资到位资金275亿元，同比增长38%，实际利用外资1.74亿美元，同比增长20%。重点建设投资10亿元的同江造船基地等装备制造业项目23个、投资5.6亿元的无菌灌装豆乳项目等绿色食品加工项目22个，一期投资20亿元的泉林秸秆综合利用以及一大批林木建材加工、可再生资源利用等项目。投资30亿元加快全市各园区基础设施建设，强化配套服务功能，推进产业项目在园区落地发展。加强重大基础设施建设，大力推进佳鹤、佳双、同集铁路扩能，抚远机场，市区西北绕城松花江二桥，哈佳客运专线，佳木斯机场迁建，同江铁路大桥，黑瞎子岛开发建设等提升城市功能的重大项目，强化区域中心城市的服务功能。着重抓好当年投资8亿元的唐人中心城市综合体等19个现代服务业项目建设，谋划并建设一批信息产业项目，提升中心城市的带动力和引领作用。

　　（二）推进现代化农业发展，加快新农村建设。加快种植业结构调整，新增水田和玉米种植面积各100万亩。抢抓农时，推广先进技术，科学田间管理，标准化种植水平达到80%以上；加快临江、引汤等8大灌区建设，保障水稻优质高效发展；农机总动力达到340万千瓦，综合机械化程度达到91%。突出典型引路，以机械化、水利化、科技化、标准化、规模化为牵动，续建8-12个10万亩以上的优势种植产业带，引导种植业向优质高效方向发展。全市粮食产量增长5%以上。结合牧草种植，加大推进畜牧生产标准化、规模化力度，畜牧业总产值达到100亿元，增长29.8%，大力发展龙头大户专业养殖，为加工业发展夯实基础；充分利用水资源优势，探索水产养殖业发展新路，水产品总产量达到5.4万吨，渔业经济达到农业总产值的5%。继续推动绿色食品基地建设，努力把佳木斯打造成全国驰名的绿色食品城。加快提高农业组织化程度，推进探索农民、农产品和市场的有效对接。依托龙头新建农业专业合作经济组织70个，提高农民增收幅度和抵制风险能力。加快推进新农村建设，完善中心城镇体系规划和基础设施建设，各县（市）区有示范、有启动、有过程，引导农民向县城、中心镇和中心村集中。进一步加强场县共建，共建面积突破500万亩，并深化共建内容。

　　（三）转变经济发展方式，提升工业经济运行质量和效益。做大做强传统优势产业。增强企业自主创新能力，推动电机、煤机、约翰迪尔、北方水泥、沃尔德电缆等优势企业扩能改造提挡升级。要强化要素集聚，延伸产业链条，促进配套企业联动发展。重点抓好核电1E级电缆材料、高效水泥等新材料产业产品研制开发。加快推进风电、生物质发电等新能源产业企业发展。依托葵花药业、多多药业等企业，做大做强中药产业，促进西药产业的二、三类新药开发。骨干企业实现新增销售收入50亿元。实施小企业示范工程，加快民营经济发展。推进完善中小企业服务平台建设，充分发挥中小企业服务平台作用，开展“走进中小企业，解决发展难题”活动，帮助企业解决生产发展中观念、管理、技术、人才、融资等问题。

　　（四）着重提升县域和城区经济，统筹区域协调发展。立足改变我市县域和城区经济总量小，支撑自身经济发展的能力弱，城镇化推进慢等状况，把发展县域和城区经济作为全市经济社会发展的重要支点。县域经济坚持走特色发展之路，突出产业立县，项目强县，培育壮大优势特色产业，实现错位差异化发展。支持县域工业做强、做实、做精，通过建立园区，强力招商引资上项目，尽快形成项目集聚，形成主导产业，全市县（市）新增规模以上企业30户，实现县域工业经济大幅增长。各县招商引资要紧紧围绕产业转移、发达地区闲资，因地制宜、突出特色，服务城市、服务大企业、服务俄远东地区。城区经济发展要紧紧依托区位条件，突出城区特点。围绕文化创意、服务外包、信息咨询、信息处理、专业培训等服务业，打造特色化、产业化功能街区。围绕餐饮、娱乐、商业及综合体等，打造中心消费区。围绕总部经济、金融保险、会展经济、楼宇经济等，打造中心商务区。围绕科研院所、大专院校人才和科技、教育优势等，打造中心科教区。充分调动办事处、社区人员工作积极性，不求形式，加强社区管理和社区建设，但求实效，全员动手，共同努力，开创区街经济发展新局面。城区经济增幅要高于全市平均水平。

　　（五）扩大对外开放的深度和广度，加快发展现代服务业。全力支持抚远黑瞎子岛开发建设和抚远的经济发展，做好抚远发展的后方基地，加快推进同江东西两港扩建改造工程，提高口岸过货能力；扎实推进佳富路升级改造和省公路应急救援中心建设；恢复佳木斯至哈巴航线，加密佳木斯至哈尔滨、上海、北京、韩国首尔航班，开通佳木斯经大连至三亚航线。抓好进出口园区建设，将出口抓加工、进口抓落地落到实处，并形成税收贡献。推进黑龙江东部商贸城，三江温州批发城等商贸项目建设。以举办好第三届中俄（佳木斯）农机产品展销洽谈会为牵动，推动会展经济全面启动。进一步完善农村商业经营网络系统，村级农家店覆盖率达到98%以上，家电下乡补贴率达到90%以上。加快旅游产业提档升级，推进香格里拉、长城、农垦大厦等星级酒店建设；加大四丰山景区、柳树岛景区等重大项目和省级、市级旅游名镇建设力度；大力发展农垦科学院采摘园等城镇周边农业观光旅游、乡村旅游、红色旅游、过境旅游；举办好各具特色的四大节庆活动。加强与友好城市文化、卫生、教育、体育交流，带动经贸合作。做好在香港、澳门，韩国、日本，加拿大、美国招商周活动，争取与法国、新西兰、乌克兰等国家有关城市缔结友好城市。以各项开放活动推动全市人民树立开放思维，敞开开放胸怀，让佳木斯融入世界经济发展格局中，以寻求快发展、大发展的机遇。

　　（六）加强城市建设和管理，提升城市承载能力。围绕区域中心城市发展目标，及时完善城市总体规划，加快地理信息系统建设，做好城区供排水、供热、燃气、道路的整体规划设计，使工程建设和管理有规可依。市区重点推进双合路、长安东路、中山路、红旗路南至四丰山路，胜利路西连鹤大公路及全线扩建慢车道等重点路段建设，继续实施区街巷道改造。各县（市）城关镇要修建好破损道路，打通断头路。要加大城乡绿化美化力度，建好绿化防护林带。市区结合内河整治，抓好内河沿岸绿化景观带、城市道路绿化带、小游园绿化建设。加大硬铺装和绿化力度，消除市区中心区及县（市）城关镇主要街路裸露土和超高土。逐步改造主要街路临街破损楼房立面，形成独特的建筑风格。做好城区亮化规划设计和实施，让大街小巷亮起来。结合楼体节能改造和小区庭院整治，建立多元融资渠道，启动旧小区改造三年计划，提高居民居住环境水平。力争创建3—4个省级示范小区。抓好沿江景观带升级改造和延伸建设，充分利用好公园、沙滩、江水等载体，让江滨亮起来，江水活起来，群众乐起来。着重抓好城市综合治理，规范牌匾广告；继续推动三废出社区工程，完善废物回收体系和旧物交易市场。进一步加大拆除违章建筑力度，坚决不让少数人损害公众利益。进一步规范建筑工地文明施工并实行一票否决。加强市区各种残土垃圾综合治理，推动清扫、清雪作业机械化、管理上水平、运转新机制，让全市干净整洁。加大市区停车场（位）规划建设，确保交通畅通。加快县（市）建制乡镇基础设施建设，促进市区、县（市）、镇协调互动发展，推进城镇化进程。着力实施“十二五”松花江流域污染防治规划项目，确保界河水质安全。

　　（七）全力拓宽财税征收和融资渠道，提升保障能力和服务水平。大力开源节流，调整和优化财源结构，促进财政收入增长。深化社会综合治税，加强重点税源、新增税源的掌控分析，充分挖掘税源潜力，应收尽收。强化零散税源征缴。规范行政审批程序，严格执行收支两条线制度，提高非税收入对公共财政的贡献水平。压缩机关行政支出，实现公务购车和用车经费、会议经费、公务接待费用、党政机关出国（境）经费预算“四个零增长”。建立健全覆盖各级财政的预算执行动态监控体系，深入推进财政科学化精细化管理，提高财政管理的效能和财政资金使用效益，全力保工资、保运转、保民生。进一步拓宽融资渠道，盘活资产，利用资源，探索一条市场化融资安全创新之路，为发展提供财政支持。继续推动小额贷款公司、村镇银行等新型金融服务体系建设。积极引进外埠银行，加快推进富锦市、桦南县等县(市)成立村镇银行。继续推进中小企业信用体系建设工作，健全融资信用担保体系，扩大市域金融机构贷款规模，力争今年新增贷款100亿元。严格监管信贷公司规范运营。为银企合作搭建平台，提高金融服务水平。

　　（八）积极推进民生工程，加快社会事业发展，努力为人民群众谋福祉。（1）加强蔬菜基地建设，总结推广市政府和农垦科学院合作引进以色列大棚技术，全市建设蔬菜基地67万亩，新增8.4万亩，延长地产蔬菜供应期。（2）完成阳光工程培训1万人，有组织转移劳动力35.5万人次，实现劳务收入25亿元。（3）推进创业型城市建设，小额担保贷款投放1亿元，扶持微小企业5000户，新增创业小老板5000人。（4）实现社会养老保险制度层面的全覆盖。探索实行低保家庭就医、上学、供热、供水、供气、公租房、法律救助等“一卡通”管理模式。建立群团、财政、各民间协会等扶助联席会议制度，让救济最大限度发挥作用。（5）加大城区现有公园绿地亮化等设施维修和保护力度，规划建设博爱主题公园和知青纪念碑。（6）开工建设一座日焚烧900吨的城市垃圾处理厂；西区污水处理厂正式运营；投资1亿元建设城区排水工程。（7）启动全市供热规划设计，规范热源热网用户三者关系，重点解决热源不足、热网不畅、小锅炉弃管问题，让群众住上暖屋子。（8）市区投资1000万元，新增燃气管线26公里，新建液化气汽车加气站2座、燃气汽车加气子站3座，新增天然气用户1万户。扩大城区和各县（市）天然气普及率。（9）城区改造棚户区60万平方米，新建廉租房1300套，公租房700套，新增租赁补贴1000户。确保2010年征收户今年年底回迁。改造农村泥草房1.5万户、120万平方米。（10）改造旧小区37个; 将弃管和管理不到位小区，按新机制成立社区物业服务站40个，除收取物业费外，政府每站年补助4万元。（11）启动江边游泳区规划建设，修建沿江锻炼跑道，在柳树岛建设大型电子屏幕和钟楼。（12）提高市区及县（市）城关镇清雪机械化率。市区主次干道清雪机械化率达80%，县（市）主次干道清雪机械化率达到60%。（13）加大教育投入，今年投入达到一般预算的20%。市财政安排500万元教育专项基金，用于校长培训、学生心理教育、学科教学研究、国际交流，让学生健康学习，获取最佳知识，力争用两年时间高考排名进入全省前三名。（14）加大校舍安全工作，今年全部完成市县（市）区D级C级校舍安全修建工程。（15）加大公办幼儿园建设支持力度；加强对民办幼儿园监管，多形式扶持民办幼儿园发展。（16）市县财政安排资金，所有城区和中心镇临街学校门前全部安装减速带、红绿灯、划上过街斑马线。城镇所有学校旱厕全部改为水厕。（17）加强学校周边的环境整治，环境秩序井然。特别对餐饮、食品销售严格管理，让学生吃得放心。公办寄宿制学校，食堂一律不准对外承包，各级财政给予相应补贴。下决心解决好学校校车问题，让孩子高兴上学，安全回家。（18）加强对大中专学校及职业学院支持力度，启动图书馆、自然标本馆等科技资源向社会免费开放，资源共享。市本级科技三项费由120万元增加到500万元，鼓励科研人员创新创业。（19）市财政安排500万元设立文化及产业发展基金，结合文化艺术团体改革，推动各种形式的文化水平提高，送文艺到基层，每年推出一部以上群众喜闻乐见的优秀文艺剧目；扶持传媒、印刷、文化手工艺品、图书等产业做精做强，提升知名度和美誉度。积极推动“赫哲族文化生态保护实验区”建设。年底前市广电大厦完成建设并投入使用。（20）积极推行三级医院领办社区卫生服务中心模式，全市市区及各县（市）要全面落实并完成社区卫生医疗中心建设并纳入财政扶持运行，方便群众，让群众满意。（21）加强特色医疗专科建设，市中心医院、佳大附属医院率先引进国内外高端人才和专家名医，让百姓不出市就能享受高水平的医疗服务。（22）市财政安排资金支持各类群众体育协会发展，探索学校、企事业、政府体育场馆免费向公众开放新机制，提高资源利用效率，提高全民健身和特色竞技体育水平。（23）市财政安排500万元设立平安佳木斯专项基金，主要用于侦破大要案奖励和见义勇为人士奖励。从今年开始全市基层派出所供热、供水、供电等基本办公经费由市县（区）两级财政分别统一安排解决。（24）城乡独生子女父母奖励费当年全部兑现，历史欠账补发20%以上。加强对老年公寓监督检查和扶持力度，让老年人享受幸福生活。（25）继续推进全市交通整治，一抓到底，形成常态化管理机制。研究解决城市公交百姓关心问题，探索佳鹤、佳双长途客运城市公交化试点，提升佳木斯中心城市作用。（26）加强市场监管，强化工商、质检、酒类、畜牧、食品药品的监管和检测，让百姓吃上放心食品，推进食品放心城建设。（27）加强安全生产监管网络，全面落实责任制，市财政投入1200万元在市区配备消防举高车，保证我市高层建筑应急安全。（28）完善突发公共事件应急预案体系，市政府和市移动公司合作，市区安装3300个探头，重点区域实现全覆盖，建立应急指挥中心平台，并提高对城市管理和社会治安，防灾减灾监督能力。积极化解积案和群众反映的诉求，市政府每月一次集中听取重点和难点问题，决不让能解决的问题久拖不决。（29）加强军民合作共建，支持驻佳部队和预备役部队建设，充分发扬我市军民一家的传统，争创全国双拥模范城“八连冠”。（30）推进佛光寺修缮、完成清真寺复建工程，全面做好民族宗教工作。

　　（九）加强政府自身建设，进一步提高政府的公信力和执行力。强化政府工作人员责任意识、落实意识、服务意识、法律意识，努力建设创新、务实、高效、廉洁、服务型政府。强化责任体系，规范行政执法行为，落实违法行政责任追究，坚决查处失职渎职和不作为、乱作为、“中梗阻”、互相掣肘、推诿扯皮现象，不断提升政府公信力和执行力。全面加强政府效能建设，年底前力争新服务中心大厅建成投入使用，市办公中心全面推行电子政务，提高工作效率。严格执行重大决策集体研究、专家咨询、社会公示等制度，确保行政权力运行公开透明、依法规范。切实转变工作作风，深入基层，认真解决人民群众反映的热点难点问题，坚决维护广大人民群众的根本利益。大力倡导用心想事、谋事、干事、干成事的良好风气，保持奋发有为、昂扬向上的精神状态，提升干事创业的劲头。勇于面对复杂局面，敢于触碰矛盾，切实解决问题。自觉执行市人大及其常委会制定的重大事项报告制度，主动接受人大法律和工作监督、市政协的民主监督以及社会监督、舆论监督、群众监督，做好审计监督。认真办理人大代表议案、建议和政协委员提案。严格执行党风廉政建设责任制，推进惩治和预防腐败体系建设。治庸治懒，严肃查处吃拿卡要，以权谋私等腐败现象，规范政府工作人员从政行为，树立敬业勤政、为民务实、廉洁高效的人民政府形象。

　　金戈铁马待征鼓，只争朝夕启新程。各位代表，在省委、省政府和市委的正确领导下，在你们的信任和重托中，我们充满信心，紧紧依靠和团结全市人民，励精图治，开拓进取，为建设富裕、文明、和谐、幸福佳木斯而努力奋斗！佳木斯的明天一定会更加美好！
